# The effects of N‐addition on litter mixture effects depend on decomposition time: A case from mixed‐litter decomposition in the Gurbantunggut Desert

**DOI:** 10.1002/ece3.10377

**Published:** 2023-08-07

**Authors:** Hong‐Mei Zhao, Wei‐Jun Yang, Jun‐Hui Cheng, Gang Huang, Yu‐Tong Hu, Cong‐Juan Li, Jian‐Dong Sheng

**Affiliations:** ^1^ Xinjiang Key Laboratory of Soil and Plant Ecological Processes, College of Resources and Environment Xinjiang Agricultural University Urumqi China; ^2^ College of Agronomy Xinjiang Agricultural University Urumqi China; ^3^ Institute of Geography Science Fujian Normal University Fuzhou China; ^4^ National Engineering Technology Research Center for Desert‐Oasis Ecological Construction, Xinjiang Institute of Ecology and Geography Chinese Academy of Sciences Urumqi China

**Keywords:** desert ecosystems, litter decomposition, litter quality, mass loss, mixture effects, nitrogen addition

## Abstract

Changes in nitrogen (N) deposition and litter mixtures have been shown to influence ecosystem processes such as litter decomposition. However, the interactive effects of litter mixing and N‐deposition on decomposition process in desert regions remain poorly identified. We assessed the simultaneous effects of both N addition and litter mixture on mass loss in a litterbag decomposition experiment using six native plants in single‐species samples with diverse quality and 14‐species combinations in the Gurbantunggut Desert under two N addition treatments (control and N addition). The N addition had no significant effect on decomposition rate of single‐species litter (expect *Haloxylon ammodendron*), whereas litter mass loss and decomposition rate differed significantly among species, with variations positively correlated with initial phosphorus concentration and negatively correlated with initial lignin concentration. After 18 months, the average mass loss across litter mixtures did not overall differ from those predicted from single species either in control or N addition treatments, that is, mixing of different species had no non‐additive effects on decomposition. The N addition, however, did modify the direction of mixture effects and interacted with incubation time. Added N transformed synergistic effects of litter mixtures to antagonistic effects on mass loss after 1 month of decomposition, while transforming neutral effects of litter mixture to synergistic effects after 6 months of decomposition. Our results demonstrated that initial chemical properties played an important role in litter decomposition, while no effects of litter mixture on decomposition process in this desert region. The N addition altered the litter mixture effects on mass loss with incubation time, implying that increased N deposition in the future may have profound effects on carbon turnover to a greater extent than previously thought in desert ecosystems.

## Graphical abstract

This paper demonstrated that the initial chemical properties played an important role in litter decomposition, while no effects of litter mixture on decomposition process in this desert region. The N addition altered the litter mixture effects on mass loss with incubation time, implying that increased N deposition in the future may have profound effects on carbon turnover to a greater extent than previously thought in desert ecosystems.

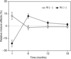

## 1 INTRODUCTION

Litter decomposition is a key process in the carbon (C) budget and nutrient cycling in terrestrial ecosystems and is closely linked with important ecosystem attributes such as productivity, community structure, and food‐chain dynamics (Adolfo et al., 2017; Parton et al., 2007). Decomposition processes and determinants based on individual species decomposition have been extensively studied and are controlled by multiple factors, including litter quality, climate parameters, and biotic factors (Austin & Vivanco, 2006; Bradford et al., 2016; Parton et al., 2007). These studies ignored interactions among litter of different species, which strongly affect decomposition rates in diverse communities (Gartner & Cardon, 2004).

In natural plant communities, the litter layer usually comprises multiple species that decompose together. Mixtures of different species can result in active translocation and leaching of nutrients and inhibitory compounds between litter species (Handa et al., 2014) and in the modification of microenvironment conditions (Makkonen et al., 2013)—changes in both would likely influence decomposition rates and nutrient transfer (Butenschoen et al., 2014; Santonja et al., 2015). Previous studies have found that synergistic non‐additive effects were common for litter mixture, sometimes mass loss in mixtures exceeds expected decomposition as high as 65% (Barantal et al., 2014; Gartner & Cardon, 2004; Handa et al., 2014). However, neutral or even antagonistic effects have also been observed, depending on the component species and the environmental context (Gripp et al., 2018; Wardle et al., 1997; Zhang et al., 2013). These conflicting results imply that it is hard to accurately predict decomposition and ecological process at community level, as decomposition patterns cannot be evaluated on the basis of single‐species decomposition dynamics. Accordingly, data from single‐species litter decomposition might be biased for estimating C and nutrient fluxes at ecosystem level (Hoorens et al., 2010; Njoroge et al., 2022); especially in temperate desert, where few studies have addressed decomposition dynamics of mixed‐litter compared with temperate forest and/or grassland ecosystems (e.g., Butenschoen et al., 2014; Gartner & Cardon, 2004). Hence, further studies examining the effect of litter mixtures on decomposition processes in desert ecosystems can help us understand the underlying mechanisms of decomposition and accurately predict the C budget at the ecosystem level.

Nitrogen (N) application for agriculture has increased the rates of N deposition in arid and semi‐arid ecosystems, and this is predicted to further increase (Fenn et al., 2003; Liu et al., 2011). Notably, increased atmospheric N deposition in response to global climate change is impacting ecosystem processes such as litter decomposition (Liu et al., 2011; Schuster, 2016). The N availability typically limits organic matter decomposition in (semi) arid ecosystems (Fisher et al., 1988). Generally, N addition may alleviate decomposer N limitation by increasing soil N availability thus enhancing litter decomposition rates (Galloway & Cowling, 2002; Liu et al., 2010). However, results of N fertilization experiments have been equivocal; for example, exogenous N addition has increased mass loss (Hou et al., 2021; Wang et al., 2017), had neutral effects (Hobbie, 2005), and inhibited rates of decomposition (Tan et al., 2020; Zhang et al., 2016), depending on fertilization doses, ecosystem N status and differences in litter chemistry (Knorr et al., 2005). Consequently, lack of a strong directional effect with N addition on litter decomposition limits our understanding of the responses of litter decomposition to future climate change.

Recent studies found that the litter mixture is expected to change decomposition rates due to interactions among litter species in mixtures, such as the complementary use of nutrients and transfer between dissimilar litters (Gartner & Cardon, 2004; Handa et al., 2014). The addition of N could regulate litter species interactions, transforming neutral effects of litter mixtures to synergistic effects on mass loss (Vivanco & Austin, 2011); thus, N addition can have significant consequences for decomposition in mixed‐litter, and it is reasonable to expect that N deposition would alter leaf litter mixture effects on decomposition in (semi) arid regions. Despite their potentially important role in regulating biogeochemical cycling, far less attention has been given to the potential interactive effects of litter mixture and N addition on decomposition in (semi) arid ecosystems. Some existing studies included only a limit number of species and/or single harvests (e.g., Liu et al., 2010), but there remains a lack of understanding of the magnitude and direction of the effects of N addition and litter mixture effects on decomposition change as decomposition proceeds. Therefore, elucidating the overall responses of decomposition in mixed‐species litter to N deposition is crucial to understanding the impact of changes on the C budget and nutrient cycling under future climate change in arid land.

The Gurbantunggut Desert is a typical temperate desert in northwestern China, where vegetation coverage can be up to 40%, and litter is the most important contributor of soil C and nutrient input (Chen et al., 2009). Future climate change scenarios have predicted increasing N deposition of temperate desert in northwestern China (Liu et al., 2011). Some results have been reported the potential responses of single‐species decomposition to N deposition. However, very few studies have accounted for the interaction effects between litter mixture and N deposition, limiting our understanding of the overall N effects and its interaction with litter diversity. In this study, six native plants with diverse litter qualities and 14 species‐combinations were used to examine the simultaneous effects of both N addition and litter mixture on decomposition in a manipulative field experiment in the Gurbantunggut Desert. We hypothesized that (a) mass loss of litter mixtures deviates from expected values from single‐species litter incubation due to the interaction between dissimilar litters; (b) the mixture effects on mass loss would vary with decomposition time; (c) N addition would alter litter mixture effects on mass loss because it disrupts species interactions and that the effects change as decomposition proceeds.

## 2 MATERIALS AND METHODS

### 2.1 Study site

The study area is located in the vicinity of the Fukang Desert Ecosystem Research Station, Chinese Academy of Sciences, on the southern edge of the Gurbantunggut Desert (44°22′ N, 87°55′ E). This region has a typical continental arid climate with a dry hot summer and cold winter. The annual mean temperature is 6.6°C, annual mean precipitation is 70–150 mm and corresponding pan‐evaporation is more than 2000 mm (Xu & Li, 2006). Rainfall has strong annual and seasonal variability, with 70%–80% occurring during in the plant growth season of April–September. The soil is eolian sandy soil in Chinese Soil Classification, which is equal to Arenosols in FAO‐WRB system (IUSS Working Group WRB, 2015). The natural vegetation is composed of desert shrubs and herbaceous ground layer. *Haloxylon ammodendron* (C. A. Mey.) Bunge, *H. persicum* Bunge ex Boiss. et Buhse, *Tamarix ramosissima* Ledeb., and *Nitraria sibirica* Pall. are dominant shrubs, with coverage of ca. 30% (Huang & Li, 2017). The herbaceous ground layer is composed of spring annuals, summer annuals, and perennials, with high inter‐variation in plant canopy cover (range: 5%–30%) (Liu et al., 2016). The dominant herbaceous plants are *Schismus arabicus* Nees, *Erodium oxyrrhynchum* M. Bieb., *Eremurus inderiensis* (M. Bieb.) Regel, *Salsola nitraria* Pall., *Suaeda salsa* (Linn.) Pall., *Salicornia europaea* Linn., *Ceratocarpus arenarius* Linn., *Karelinia caspia* (Pall.) Less., and *Sophora alopecuroides* Linn. (Huang & Li, 2017; Liu et al., 2016).

### 2.2 Experimental design and treatments

The experiment used a randomized block design with two N treatments – control (no fertilization, N−) and N addition (N+, 10 g N m^−2^ year^−1^) – with each treatment replicated five times. A total of 10 plots were arranged on flat inter‐dune ground, with an area of 3 × 3 m for each plot and adjacent plots were separated by a 2‐m buffer. For the N addition treatment, urea solution was manually applied with a sprayer on October 11, 2017 with four other equal applications in 2018 (March–June), totaling 10 g N m^−2^ year^−1^. This amount of fertilizer was based on recommendations for alleviating N limitation in temperate deserts (Liu et al., 2011). Fertilizer was weighed, mixed with 2 L of water and applied to plots with a sprayer. The control plots received 2 L of water without N addition to avoid differences in moisture application.

### 2.3 Plant material

Based on plant dominance, life‐form, and major litter organs in the native community, six plant litter types were chosen for this experiment. Current‐year fallen assimilating branches of *H. ammodendron* (C. A. Mey.) Bunge (Ha) and stems of *T*. *ramosissima* Ledeb. (Ta) and leaves of *N*. *sibirica* Pall. (Ni), *K*. *caspia* (Pall.) Less. (Ka), *S*. *europaea* Linn. (Sa), and *S*. *alopecuroides* Linn. (So) were collected in August–September 2017. All litter was air dried to a constant weight in the laboratory.

Litter mixtures were constituted from the six above‐mentioned litter species. There are 20 different litter treatments with species richness in the range of 1–6, including all single‐species treatments and mixtures of two, three, four, and six species. Litter species were equally often represented within each level of litter species richness: each species was present in exactly two 2‐species, two 3‐species and two 4‐species mixtures, additionally to the single 6‐species mixture. This balanced design allowed us to disentangle the effects of species number from that of mixture composition and to explore effects of the presence/absence of each litter species within mixtures (Barantal et al., 2014).

Three subsamples were randomly chosen from each litter type (20 litterbag types) and oven‐dried for 48 h at 70°C at the time of initial deployment to determine the initial dry mass and to analyze initial tissue chemistry. Oven‐dried subsample litter was separately milled to powder using a ball mill (MM400, Retsch GmbH, Haan, Germany), and used to analyze initial C, N, phosphorus (P), potassium (K), hemicellulose, cellulose, and lignin concentrations—the initial tissue chemistries differed substantially among species (Table 1). Initial concentrations of C and N were determined using an elemental analyzer (Euro Vector EA3000, Redavalle, Italy). Initial P concentration was measured using the molybdenum blue colorimetric method (Bao, 2000). Hemicellulose, cellulose, and lignin were analyzed using the sequential extraction technique (Van Soest et al., 1991). Subsamples (0.5 g) were used to determine neutral fiber detergent, acid fiber detergent, and lignin (sulfuric acid digestion) levels with an ANKOM A200i Semiautomatic Fiber Analyzer (ANKOM Technology Corp, Macedon, NY, USA).

**TABLE 1:** Initial chemical characteristics in litters of single species (mean ± *SE*, *n* = 3).

Initial chemical composition	*Haloxylon ammodendron*	*Tamarix ramosissima*	*Nitraria sibirica*	*Karelinia caspia*	*Salicornia europaea*	*Sophora alopecuroides*
Carbon (%)	51.48 ± 0.23a	45.68 ± 0.66b	50.05 ± 0.20c	51.65 ± 0.38a	49.40 ± 0.52 cd	48.64 ± 0.05d
Nitrogen (%)	3.22 ± 0.05a	3.26 ± 0.09a	5.25 ± 0.02b	3.47 ± 0.02c	2.34 ± 0.08d	3.65 ± 0.04e
Phosphorus (%)	0.11 ± 0.00a	0.17 ± 0.01b	0.23 ± 0.00c	0.24 ± 0.00d	0.46 ± 0.00e	0.17 ± 0.00b
Potassium (%)	2.96 ± 0.05a	1.11 ± 0.03b	1.63 ± 0.20c	1.03 ± 0.02b	1.93 ± 0.02d	0.91 ± 0.02e
Hemicellulose (%)	14.77 ± 0.90ad	11.27 ± 0.59bd	9.88 ± 0.44b	10.12 ± 1.24b	23.88 ± 0.37c	13.29 ± 0.91d
Cellulose (%)	11.71 ± 1.03a	18.98 ± 0.42b	8.09 ± 0.37c	8.93 ± 0.52c	16.87 ± 0.55d	15.06 ± 0.83e
Lignin (%)	4.82 ± 0.21ad	7.05 ± 0.18b	4.47 ± 0.17 ac	6.71 ± 0.16b	4.19 ± 0.21c	5.30 ± 0.04d
C/N	15.98 ± 0.31a	14.01 ± 0.19b	9.53 ± 0.03c	14.90 ± 0.12d	21.18 ± 0.47e	13.34 ± 0.16b
Lignin/N	1.50 ± 0.08a	2.16 ± 0.02b	0.85 ± 0.03c	1.93 ± 0.05d	1.79 ± 0.04e	1.45 ± 0.01a

*Note:* Different lowercase letters indicate significant differences (*p* < .05) among species using a one‐way ANOVA with Levene's test as an a priori test for homoscedasticity.

### 2.4 Litterbag experiment

The litterbag method was used to investigate litter decomposition. Litterbags were made of nylon with size of 15 × 20 cm; the top and bottom layers consisted of 1 and 0.1 mm mesh sizes, respectively. Each litterbag was filled with 6 g of air‐dried litter, with mixtures containing equal mass proportions of the component species. One of each of the 20 different litter types (6 single‐species +14 mixtures) was randomly laid in each of the treatment's plots of all five blocks in October 2017. In total, 800 litterbags were used for the experiment (20 litter types × 2 N treatments × 5 blocks × 4 harvests). Litterbags were fixed with a short wire directly on the soil surface to prevent movement. Litterbags were retrieved after 1, 6, 12, and 18 months of decomposition and transported in individual envelopes to the laboratory. Sand and corpses of arthropods were carefully removed from litter by hand, and then oven‐dried for 48 h at 70°C to get mass dry weight. To correct for inorganic contaminants, all litter samples were milled to powder and analyzed for ash content following ignition in a muffle furnace for 4 h at 500°C.

### 2.5 Data analysis

Mass and initial litter properties were analyzed on an ash‐free dry matter basis to exclude any mass gain resulting from mineral soil in the litterbags. Mass loss was calculated as follows (Butenschoen et al., 2014): mass loss (%) = ((*M*_0_ − *M*_*t*_)/*M*_0_) × 100, where *M*_0_ and *M*_t_ are the ash‐free initial litter mass and remaining litter mass after a given time period *t*, respectively, and *t* is decomposition time (in months). Decomposition rate (*k*) was estimated by the negative exponential decay function *M*_*t*_/*M*_0_ = e^−*kt*^ (Olson, 1963), where *t* is the time in years.

Initially, two‐way analysis of variance (ANOVA) was used to analyze the effects of decomposition time, species, and their interactions on mass loss of single‐species litter, and differences in decomposition rate between N addition treatments were tested by paired t‐test. Partial least square (PLS) regression was used to quantify the relative importance of litter quality (initial contents of C, N, P, K, lignin, cellulose, hemicellulose, and ratios of C/N and lignin/N) for mass loss and decomposition rate of single‐species leaf litter using the R package “plsdepot” (Sanchez, 2012). We used PLS regressions instead of multiple linear regressions because PLS regression is based on the linear conversion of a large number of predictors to a small number of orthogonal factors and eliminate multi‐collinearity between predictors (Butenschoen et al., 2014; Carrascal et al., 2009). The explanatory power of each predictor in the model was estimated by the variable of importance of projection (VIP), with VIP > 1 indicating a significant effect on the dependent variable. Model strength was assessed by the explained variance (*R* ^2^ *Y*) and the model prediction power (*Q* ^2^).

The expected mass loss (*E*) in litter mixtures was calculated as follows (Gartner & Cardon, 2004; Lecerf et al., 2007):

$$E\left(\%\right)=\frac{\sum_{i=1}^{n}\left(M_{i}\times O_{i}\right)}{\sum_{i=1}^{n}M_{i}}\times 100$$

where *M*_*i*_ is the initial ash‐free dry mass of litter species *i* and *O*_*i*_ is the observed percentage of litter ash‐free dry mass loss of species *i* in the single‐species litterbag at each harvest.

Paired Student's *t*‐test on data pooled across the sample dates was used to test whether observed and expected mass loss in individual mixed‐species litter bags differed between the control and N addition treatments. To detect non‐additive effects on litter mass loss, the relative mixture effects (RME) for each individual litterbag at each harvest was calculated as the ratio of [(observed − expected)/expected)] mass loss (Wardle et al., 1997). One‐sample Student's *t*‐test on data pooled across all litter mixtures at each sampling was used to test whether RME significantly differed from zero in the control and the N addition treatments. Also, we used one‐sample Student's *t*‐test for each type of litter mixture (in the control and in the N addition treatments) to test whether RME significantly differed from zero at each sampling. Values of RME not different from zero indicate additivity, whereas positive and negative values suggest synergistic and antagonistic effects on decomposition, respectively.

ANOVAs (type І), followed by Tukey tests, were used to assess the effects of litter diversity (decomposed in species richness and species composition), time, N treatments and their interactions and blocks on RME (Santonja et al., 2015). To investigate the effects of individual litter species in the mixtures, combined with incubation time and N fertilization, we used an ANOVA with the presence/absence of each species in mixtures, time, N treatments and their interactions and blocks as factors (Santonja et al., 2015). We initially included all interactions among species presence/absence, time, and N treatments in model; this was then removed from the model and the analysis run again if the interactions were non‐significant (*p* > .05). Multiple comparisons of significant interactions were assessed with Tukey's post hoc test, and differences between including a given litter species of all mixtures and without this species were tested by paired *t*‐test. All datasets used Shapiro–Wilk to test normality, and no data transformations were conducted. Statistical analyses were performed using R software (R Development Core Team). Significance was evaluated in all cases at *p* = .05.

## 3 RESULTS

### 3.1 Single‐species leaf litter

**FIGURE 1:**
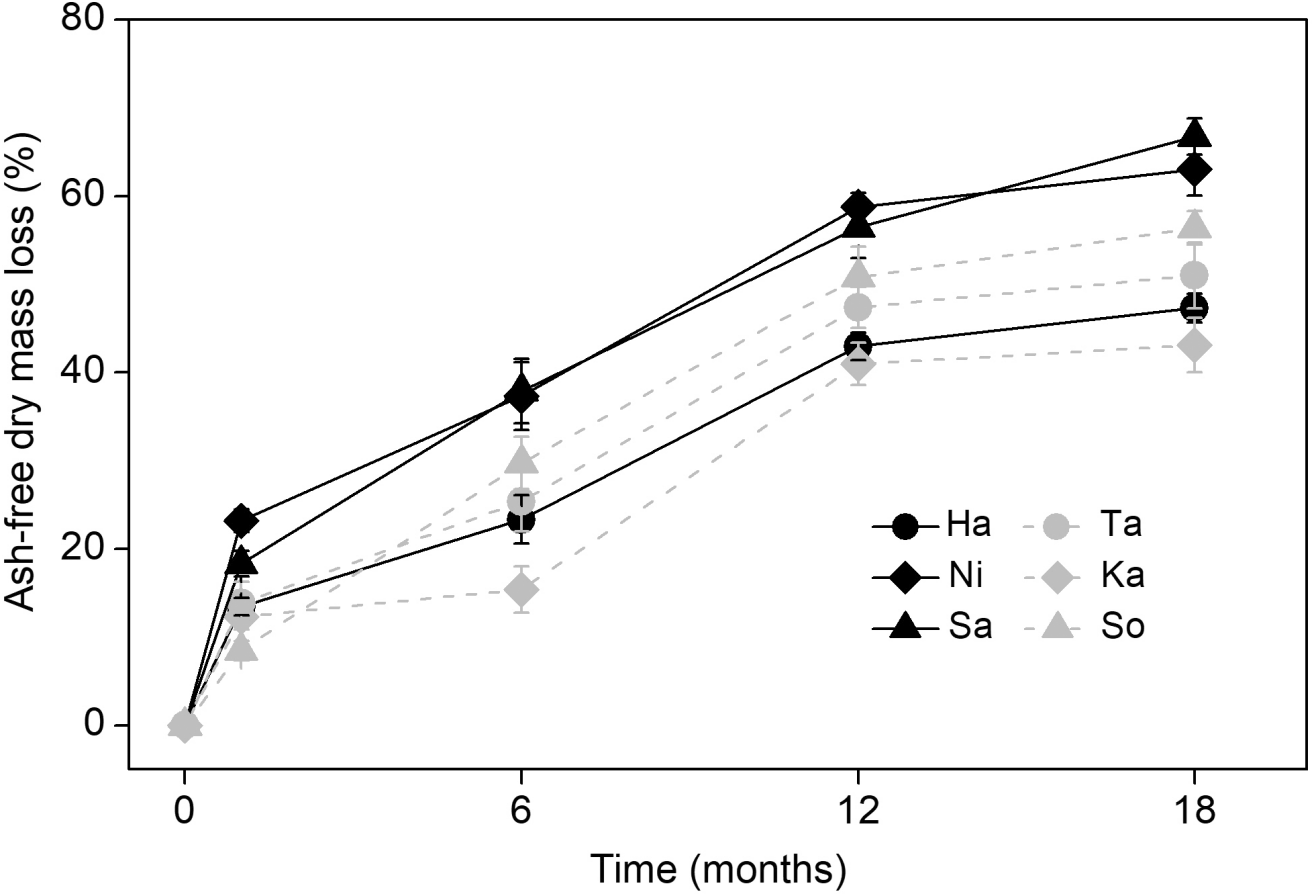
Mean ash‐free mass loss (% initial mass) of single‐species litter as affected by time after incubation for 18 months in the field.

**FIGURE 2:**
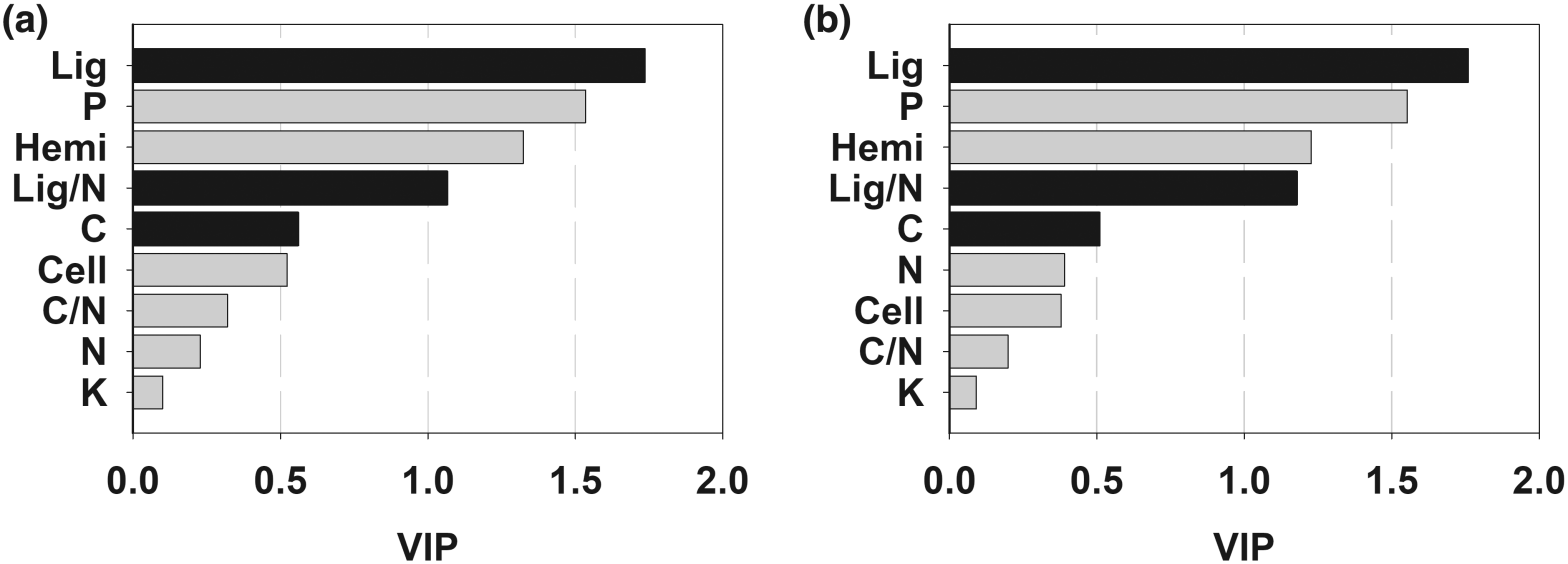
Explanatory power of initial contents of carbon (C), nitrogen (N), phosphorus (P), potassium (K), lignin (Lig), cellulose (Cell), hemicellulose (Hemi), and ratios of C/N and lignin/N (Lig:N) expressed as variable importance for projection (VIP) obtained by PLS regression analysis for mass loss (a) and decomposition rate (b) of single‐species litter (after 18 months). Gray bars indicate positive and black bars negative correlation of initial chemical composition with mass loss and decomposition rate according to standardized regression coefficients.

Decomposition time (*F*_3, 96_ = 308.78, *p* < .001) and species (*F*_5, 96_ = 30.10, *p* < .001) had significant main effects on litter mass loss. Mass loss of single‐species litter ranged from 43.11% (Ka) to 66.76% (Sa) after 18 months of decomposition (Figure 1). The PLS regression model including all initial chemical composition explained only 34% of variation of mass loss among species after 18 months. Mass loss was positively correlated (*p* < .01) with initial P and hemicellulose contents and negatively correlated (*p* < .05) with initial lignin content; other variables had low explanatory power and did not significantly correlate with mass loss (Figure 2a).

**FIGURE 3:**
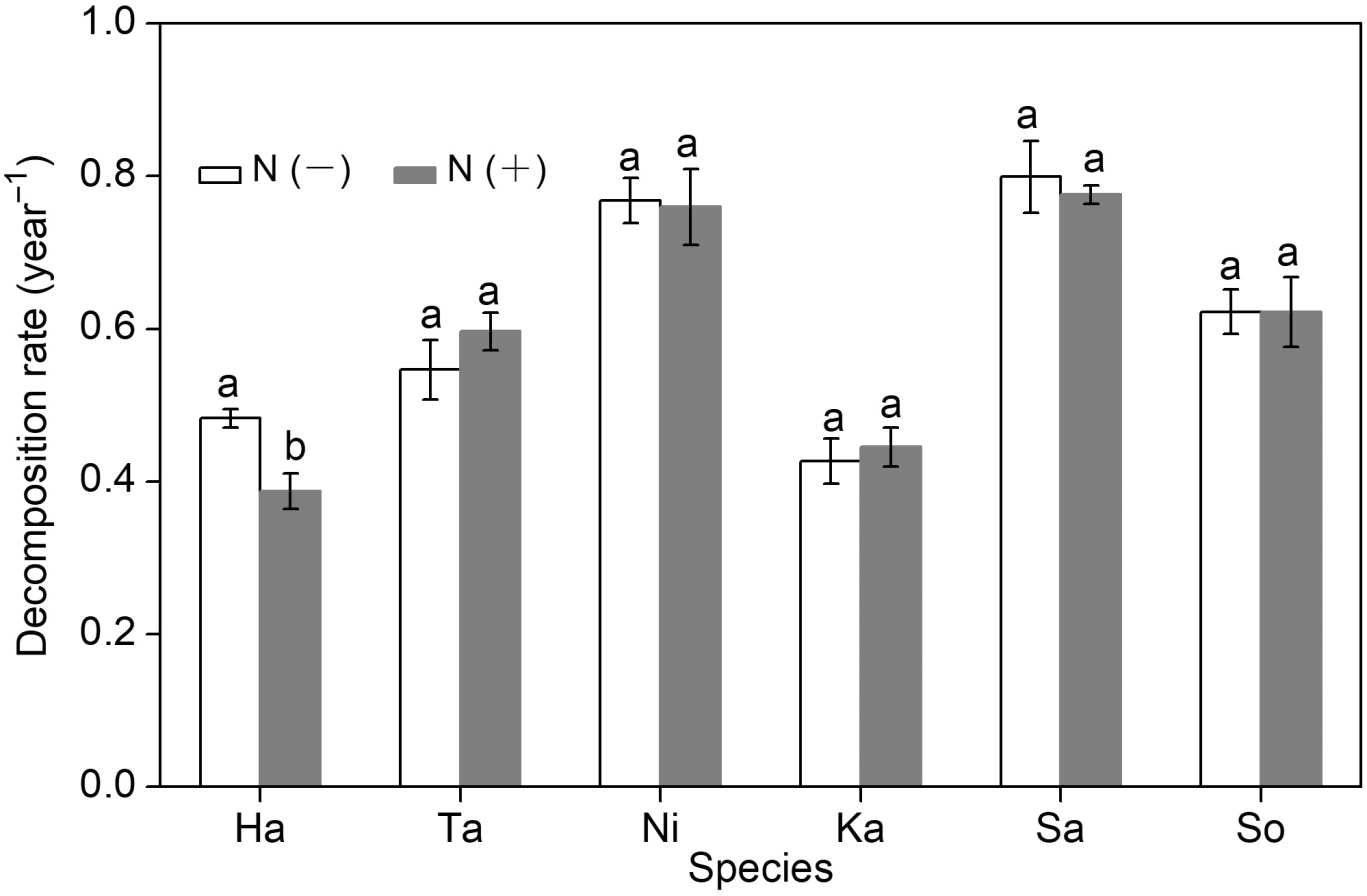
Decomposition rate (*k*) of single‐species litter under different N treatments. Different small letters denote a significant difference in *k* between N treatments at *p* < .05.

Litter decomposition dynamics was well fitted by negative exponential regression (*R* ^2^ > .86, *p* < .01). The N treatment had no significant impact on decomposition rate (*k*‐value) of single‐species litter expect for Ha, while there were strong differences in *k* among species (*F*_5, 48_ = 43.77, *p* < .001), and mean *k* was highest for Sa in the control and lowest for Ha in the N addition treatment (Figure 3). This PLS model including all initial chemical composition explained 44% of variation of *k*. Again, differences in *k* among species were positively correlated (*p* < .01) with the initial P and hemicellulose contents, and negatively correlated (*p* < .01) with initial lignin content; other variables contributed little to the regression model (Figure 2b).

### 3.2 Leaf litter mixtures

**FIGURE 4:**
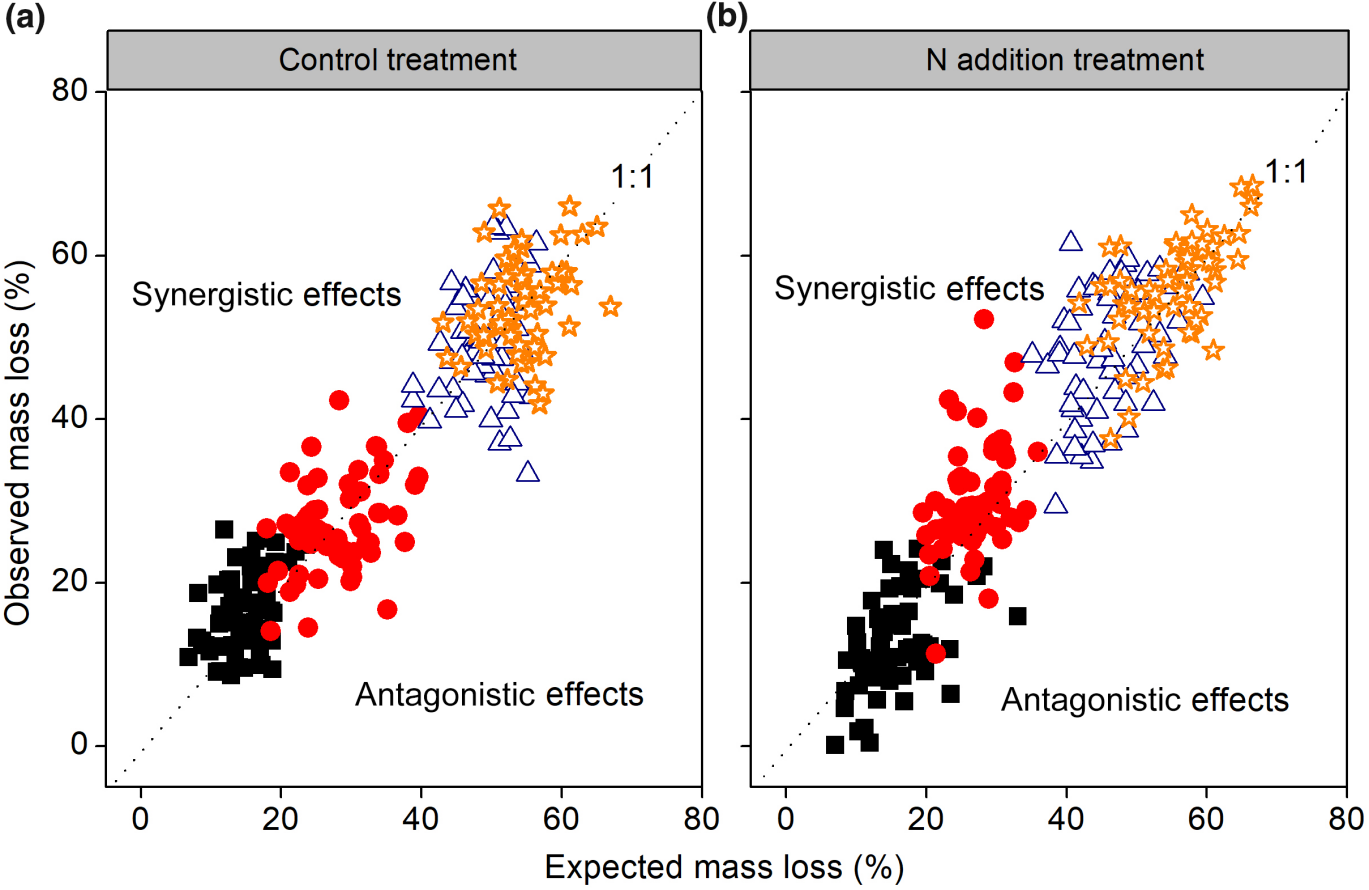
Observed mass loss of litter mixture as a function of expected mass loss (calculated as the average mass loss based on component species decomposed singly) under control (a) and nitrogen (N) addition treatments (b) in the Gurbantunggut Desert. The black symbols represent litter samples collected at 1 month; red, samples collected at 6 months; blue, samples collected at 12 months; and orange, samples collected at 18 months. The 1:1 line defines identical observed and expected mass loss values. Each point represents an individual litterbag.

**FIGURE 5:**
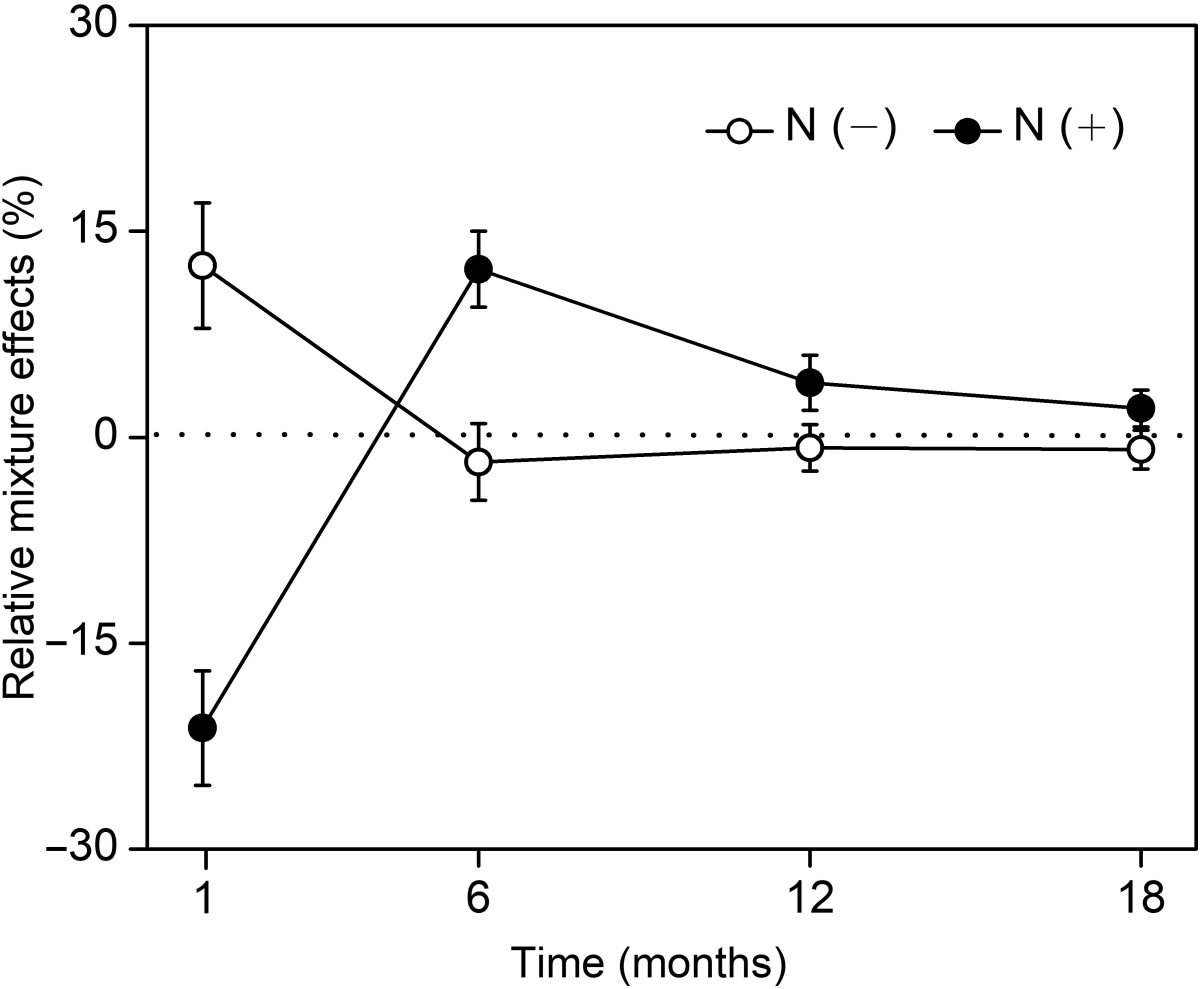
Relative mixture effects on mass loss of all litter mixtures during the decomposition process. Relative mixture effects were calculated as the ratio of [(observed − predicted)/predicted] mass loss. Positive deviations from zero indicate synergistic effects and negative deviations indicate antagonistic effects of litter mixtures.

**FIGURE 6:**
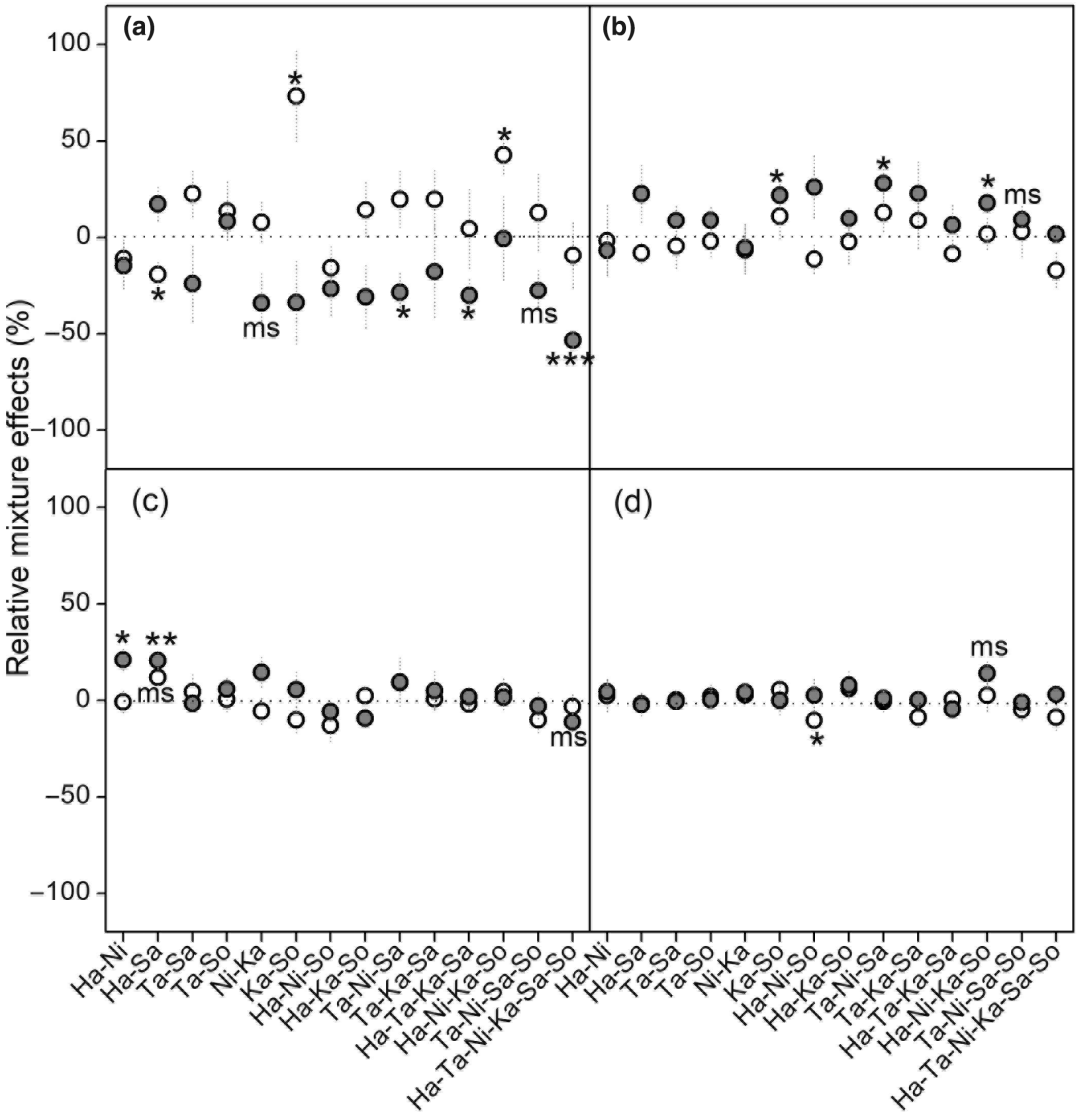
Relative mixture effects, calculated as the ratio of [(observed − expected)/expected)] mass loss, for 14 different litter mixtures incubated for 1 (a), 6 (b), 12 (c) and 18 (d) months under control (white circles) and nitrogen addition (gray circles) treatments. Asterisks denote a significant relative mixture effect, that is, a significant deviation from 0 (*p < .05, **p < .01, ***p < .001); “ms” denotes marginally significant values (.05 < *p* < .1).

Species richness and composition all significantly affected the RME, but no significant interactions between both terms and decomposition time were observed (Table 2). The overall mass loss across all litter mixtures did not significantly differ from expected values calculated from the component species single‐species decompositions when averaged over time, so there was no overall mixing effect both in control (Figure 4a) and N addition treatments (Figure 4b). However, the strength and direction of the litter mixture effect varied depending on decomposition time (Table 2; Figure 5). In the control plot, mass loss averaged across mixtures was significantly faster than expected according to the single‐species incubations after 1 month (mean RME = +12.50%, *t*_69_ = 2.73**), and did not deviate from expected mass loss after 6, 12, and 18 months (Figure 5). Specifically, at first sampling, only mass loss of the Ka–So and Ha–Ni–Ka–So was significantly faster than expected by 7.18% and 5.79%, respectively (Figure 6).

**TABLE 2:** ANOVA of the effects of litter diversity [decomposed into species richness (Sr) and species composition (Sc), time (T), nitrogen treatments (N)] and their interactions on litter relative mixture effects.

	Relative mixture effect		
	*Df*	*F*‐value	*p*‐Value
Sr	3, 444	5.18	<.01
Sc	10, 444	2.64	<.01
T	3, 444	4.41	<.01
N	1, 444	2.53	.112
Sr × T	9, 444	1.84	.058
Sc × T	30, 444	1.13	.289
Sr × N	3, 444	0.20	.895
Sc × N	10, 444	2.50	<.01
T × N	3, 444	31.33	<.001
Sr × T × N	9, 444	0.70	.705
Sc × T × N	30, 444	1.74	.010
Block	4, 444	2.60	.036

Overall, N treatment had no effect on RME, but significantly interacted with decomposition time and species composition (Table 2), implying that the influence of N supply varied with decomposition time and was dependent on litter species composition (Figure 6). Again, addition of N modified the RME according to decomposition time (Figure 5). When N was added, the average RME across all litter mixtures shifted to clearly antagonistic effects after 1 month (mean RME = −21.18%, *t*_69_ = −5.07***), particularly for Ta–Ni–Sa, Ha–Ta–Ka–Sa and 6‐species litter mixtures, in which RME were significantly lower than zero (Figures 5 and 6). After 6 months of decomposition there was a significant synergistic mixing effect, which tended to decrease with time and was not present after 18 months (mean RME = 2.09%, *t*_69_ = 1.57, *p* > .05; Figure 5).

**FIGURE 7:**
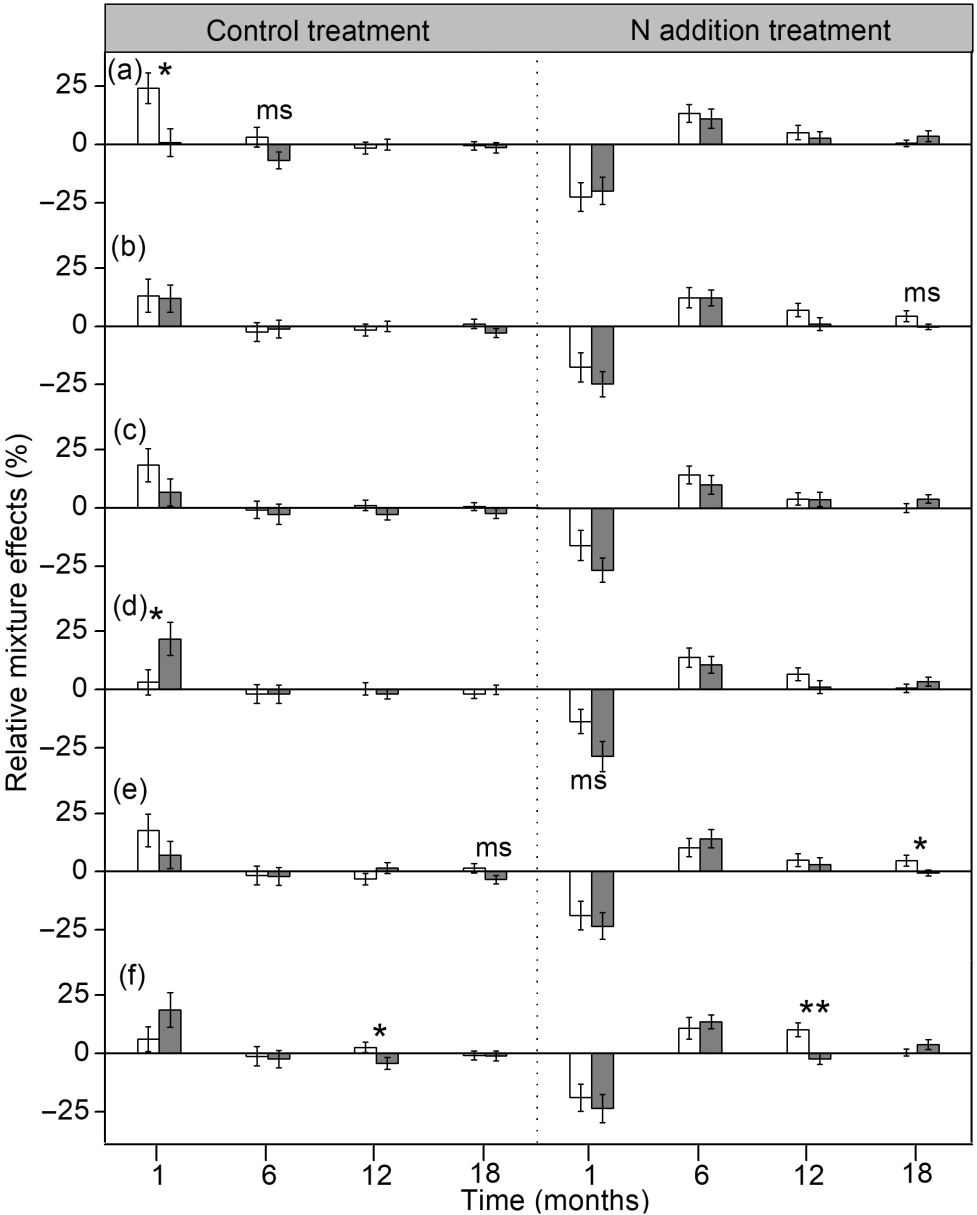
Effects of absence (white bars)/presence (gray bars) of *Haloxylon ammodendron* (a), *Tamarix ramosissima* (b), *Nitraria sibirica* (c), *Karelinia caspia* (d), *Salicornia europaea* (e) and *Sophora alopecuroides* (f) on the relative mixture effects incubated for 1, 6, 12, and 18 months in the control and nitrogen (N) treatments. Asterisks denote significant differences between absence and presence of litter species in each sampling date (*p < .05, **p < .01), and “ms” denotes marginally significant values (.05 < *p* < .1).

Litter mixture effects were also influenced by the absence/presence of Ha, Ta, and Ni litter (Table 3). Significant differences among mixtures with and without Ha and Ni litter were observed, with the absence of Ha and/or Ni litter increasing litter mass loss. By contrast, on average, mass loss of mixtures without Ta did not deviate from expected mass loss. Although fertilization with N only did not change the RME for mass loss, the effects of N supply were influenced by the presence of Ha and Ka leaf litter, and again this effect varied with decomposition time (Table 3; Figure 7). In control plots, mixtures without Ha and those containing Ka significantly increased mixture effects after 1 month but N fertilization tended to decrease mixture effects. The presence of Ha marginally significantly decreased RME from +3.18% for without Ha to −6.77% in control plots but tended to increase mixture effects with N fertilization after 6 months of decomposition, and mixture effects were independent of the presence of Ha and Ka thereafter. However, without N, the mixture effects were lower when So was present than in its absence, whereas absence of So substantially increased RME with N fertilization at the third harvest after 12 months, and after 24 months the same trend was also observed for the presence of Sa litter (Figure 7).

**TABLE 3:** ANOVA of the effects of the presence of each litter species, time, nitrogen treatments and their interactions on litter relative mixture effects.

	*Df*	*F*‐value	*p*‐Value
*Haloxylon ammodendron* (Ha)	1, 525	4.25	.040
*Tamarix ramosissima* (Ta)	1, 525	5.85	.016
*Nitraria sibirica* (Ni)	1, 525	4.00	.046
*Karelinia caspia* (Ka)	1, 525	0.21	.650
*Salicornia europaea* (Sa)	1, 525	0.01	.905
*Sophora alopecuroides* (So)	1, 525	0.00	.987
Time (T)	3, 525	4.21	<.01
Nitrogen treatments (N)	1, 525	2.42	.121
Ha × T	3, 525	1.88	.132
Ka × T	3, 525	0.71	.549
So×T	3, 525	2.59	.052
Ha × N	1, 525	4.76	.030
Ka × N	1, 525	8.16	<.01
T × N	3, 525	29.90	<.001
Ha × T × N	3, 525	2.72	.044
Ka × T × N	3, 525	5.40	.001
Block	4, 525	2.48	.043

*Note:* An initial model was computed including all interactions among species, time, and nitrogen treatments. Only interactions that accounted for significant variation in relative mixture effects were kept in the final model.

## 4 DISCUSSION

### 4.1 Single‐species litter

At the regional scale, litter quality, particularly initial concentration (and ratios) of nutrients, and recalcitrant components (e.g., lignin), is responsible for the variations of decomposition rate among species (Jiang et al., 2014; Liu et al., 2018). In the present study, pronounced differences in litter mass loss and decomposition rate among the six species were identified (Figures 1 and 3). The observed interspecific variations in decomposition rate were partly explained by initial concentrations of P and lignin (Figure 2). For instance, the Ni and Sa had higher P and lower lignin concentrations and decomposed faster than other species with lower P and higher lignin concentrations. These results are consistent with previous studies highlighting the importance of litter quality on decomposition, demonstrating significant positive correlations of litter decomposition with P concentration (Liu et al., 2018) and negative correlations with lignin concentration (Butenschoen et al., 2014). It is, however, worth noting that initial hemicellulose concentration was positively correlated with decomposition rate and mass loss after 18 months. This is a surprising result, as it is generally thought that the structural compounds (e.g., cellulose, hemicellulose, and lignin) making up plant cell walls have a negative (instead of a positive) effect on litter decomposition rate (Vaieretti et al., 2013). We speculate that the presence of Sa might have been the cause for the positive relationship between hemicellulose and litter decomposition in our study, because Sa contained a high initial level of hemicellulose (23.88%) with high decomposability. If Sa was excluded from the PLS analyses, initial hemicellulose had no significant effect on litter decomposition rate. Thus, considering the species‐specific and limited data used in this analysis, the conclusion concerning the hemicellulose–decomposition relationship should be considered with caution.

### 4.2 Litter mixture effects

In the present study, under ambient conditions, observed litter mass loss did not differ overall from the expected values when averaged over time, so there was no overall mixing effect (Figure 4). This was inconsistent with our first hypothesis and contrary to findings of earlier studies that synergistic and antagonistic non‐additive effects on mass loss prevailed in terrestrial ecosystems (Barantal et al., 2014; Gartner & Cardon, 2004). Based on data collected at each sampling date, a significant synergistic effect averaged across all litter mixtures after 1 month of decomposition was observed in control plots (Figure 5), while in most cases (11 out of 14 litter mixtures) the difference between observed and expected did not significantly differ from zero, therefore showing only additive effects (Figure 6). Differing from our result, the mixing of leaf litter strongly and consistently accelerated mass loss at the initial phase of decomposition in wet areas (Butenschoen et al., 2014; Santonja et al., 2015). This may arise because of the different physical and biotic factors that affect decomposition dynamics in dry and wet regions. In our study, these inconsistent and relatively weak effects of litter mixing on mass loss may be for two reasons. First, the extremely low water availability in desert regions probably retarded species interactions (Schuster et al., 2017), leading to species identity outweighing the effects of interactions among species on decomposition in our study, that is, the individual effects of species were additive for overall litter decay (Ball et al., 2008). Second, based on the resource‐complementarity mechanism for synergistic interactions, nutrient transfer among dissimilar litter species by leaching or microbial processes was responsible for faster decomposition in litter mixtures (Handa et al., 2014; Lummer et al., 2012). Considering that nutrient transfer within litter mixtures is stoichiometrically controlled (Lummer et al., 2012), we speculate that due to a lack of relative availability of additional resources like C required by microbial decomposers (Handa et al., 2014), the litter mixing did not accelerate or decelerate mass loss of most decomposing mixtures. However, after 1 month of incubation, synergistic and antagonistic mixture effects were observed in certain combinations under control treatment (Figure 6)—probably because presence of particular species in leaf mixtures influenced their decomposition within the mixture. This is consistent with the results of Wardle et al. (1997), who found that both synergistic and antagonistic effects of litter mixing occurred depending on the component species. In our study, synergistic interactions were observed in the 2‐ and 4‐species mixtures containing Ka and So at the 1‐month collection point. Due to its higher lignin content, Ka had the lowest decomposition rate in single‐species incubations, and the presence of Ka significantly increased mixture effects (Figure 7), indicating that nutrient translocation among dissimilar litter species may be effective for some species combinations. Presumably microorganisms captured nutrients from neighboring litter to meet the requirements of the different components of the decomposer community, accelerating mass loss of litter mixtures (Handa et al., 2014; Lummer et al., 2012). Conversely, antagonistic interactions were found in the Ha*–*Sa mixture, consistent with results of some other studies (Smith & Bradford, 2003; Wardle et al., 1997). The reasons for such antagonistic effects at the 1‐month point remain unclear but may be due to the mixing of different species' leaves changing the physical structure of litter by increasing the structural heterogeneity within the bags; this change may hamper effective functioning of specialist decomposers in heterogeneous litter (Smith & Bradford, 2003). Also, other mechanisms, such as changes of microclimate condition (Makkonen et al., 2013) and shifts in microbial biomass and functionality (Chapman et al., 2013), may be responsible for non‐additive effects. Irrespective of specific mechanisms involved, litter mixture effects would show a different pattern when species combinations were changed. However, as we did not directly measure decomposer community composition or nutrient dynamics in litter mixtures at the initial stage of decomposition, further research is needed to clarify this issue, to finally assess the contribution of each of the many possible mechanisms to the observed litter mixture effects.

Consistent with recent findings (Lecerf et al., 2011; Njoroge et al., 2023; Santonja et al., 2015; Wardle et al., 1997) and our second expectation, our results also emphasized that the litter mixture effects on decomposition varied with time (Table 2). Generally, the observed mixture effects were not static, and synergistic and antagonistic effects occurred frequently in the same litter mixtures with prolonged decomposition time, which could result from changes of microclimate, litter quality and decomposer community (Butenschoen et al., 2014). In our study, synergistic effects disappeared after the 1‐month point and changed to additive effects (Figure 5). One possible explanation is that, over time, the beneficial effects of microenvironmental changes on litter mass loss might offset by the inhibitory effects of decreasing litter quality. Previous studies have shown that changes in the decomposition environment and litter quality along a plant diversity gradient may have balanced each other out (Vogel et al., 2013), which helped to explain apparent additive effects in later stages of decomposition. Furthermore, key species traits (e.g., lignin) perhaps became more important compared to species interactions and controlled the later stages of mixed‐litter decomposition, which might cause the litter mixture effects to disappear. Unfortunately, our study did not address a comprehensive explanation for the litter mixture effects changing with time. What seems certain is that, at our study site, incubation time and the presence of specific species were all important factors influencing decomposition of mixed litter. Variations of litter mixture effects changed with time and species composition and may have substantial long‐term impacts on biogeochemical cycling.

### 4.3 N effects on litter decomposition and RME

In this study, the N addition does not change or negatively affect the litter decomposition rates for six single‐species (Figure 3), supporting the results of previous studies (Hobbie, 2005; Knorr et al., 2005; Tan et al., 2020; Zhang et al., 2016). However, several studies also found that N addition to an N‐limited ecosystem commonly accelerated the litter mass loss (Hou et al., 2021; Wang et al., 2017). The differences in mass loss responses to N addition among studies may be related to initial litter quality, site differences or biotic differences in soil and plant communities (Hou et al., 2021; Knorr et al., 2005; Zhang et al., 2016). Previous studies found that substrate quality influences the sensitivity of litter decomposition rate to exogenous N addition (Knorr et al., 2005; Tan et al., 2020; Wang et al., 2017). For instance, Kwabiah et al. (1999) found that the higher the total N or P of litter, the less significant were effects of inorganic N fertilizer addition on litter decomposition processes. In our study, the initial N concentration (2.34%–5.25%) of litter was higher than in other studies (~0.5–3.9%; Kwabiah et al., 1999; Liu et al., 2018; Zhang et al., 2016). Thus, we speculate that the supply of nutrients from the decomposing substrates may be sufficient to satisfy the decomposer demand for resources, which may be one reason for the lack of response of most single‐species decomposition to N addition. Moreover, there was no significant correlation between decomposition rate and initial N content and C/N ratio (Figure 2), implying that N was not a limiting factor for leaf decomposition (Hobbie, 2005), which could lead to reducing the response of decomposition to N addition. However, it is worth noting that N addition had inhibitory effects on the decomposition rate of Ha (Figure 3), also suggesting that the responses of decomposition to N addition were species‐specific and may have several causes. First, exogenous N might bind with products of lignin degradation to form a more recalcitrant fraction, and therefore slow decomposition (Berg & McClaugherty, 2003). This may not be the case in this study because higher N inhibitory effects were not observed for litter with high lignin content (e.g., Ta and Ka). Second, if other nutrients, such as P, become limiting for decomposer microbes (Hobbie & Vitousek, 2000), a high N inhibitory effect should be expected for litters with low P. Similarly, added N did inhibit decomposition of Ha with a low initial P concentration (0.11%), but not for other litter with high P (0.17%–0.46%; Figure 3), and makes P‐limitation of decomposition plausible after N addition in this study. Additionally, exogenous N addition during decomposition probably evoked litter‐specific responses from the microbial communities associated with each litter type due to differences in inherent quality characteristics of each plant material (Jiang et al., 2014; Tan et al., 2020). For Ha litter, N addition might change microbial community composition that results in decreased decomposition rate. It is well established that increased N leads to a reduced microbial biomass or oxidative activity of many enzymes (Tan et al., 2020; Weand et al., 2010), which indirectly support our results.

Consistent with our third hypothesis, results showed that the addition of N significantly altered the direction of litter mixture effects and that this N effect changed as decomposition proceeded (Table 2). Interestingly, synergistic effects of litter mixture on mass loss transformed into antagonistic effects under N addition conditions after 1 month of decomposition, and neutral effects of litter mixtures were transformed to synergistic effects after 6 months of decomposition (Figure 5). This suggest that incubation time significantly affected the responses of litter mixture effects to N addition. Previous studies on the effect of N addition to litter mixture effects reported conflicting results. For example, in Hulun Buir Meadow Steppe, low N addition altered the mixture effect of litter decomposition and transformed neutral effects of litter mixtures to antagonistic effects on mass loss (Zhang et al., 2013). In a Patagonian Forest, there were synergistic effects of N addition on mass loss of a three‐species litter mixture (Vivanco & Austin, 2011). Differing from our result, these authors did not observe any synergistic effects without N addition, but since they only assessed one litter mixture it is difficult to generalize the mechanism underlying the mixture effect responses to N addition from these results. In an undisturbed Amazonian rainforest using six tree species, N addition reduced the synergistic effects on decomposition since N supply probably compensated for external resource availability (Barantal et al., 2014). However, unlike our study, they did not assess the effects of N addition on the earlier and later litter mixing effects due to having just one sampling point at 158 days. Here, we demonstrate that incubation time was a key factor to capturing the response of litter mixture effects to N addition. Under future N deposition scenarios, variations of litter mixture effects with time may profoundly affect C budgets in desert ecosystems. Notably, however, we acknowledge that N addition overall did not result in significant changes to the effect of litter mixture (Figure 4), implying that litter mixing may have only a small long‐term effect on the decomposition process with future N deposition, because the positive and negative effects of litter mixture may compensate for each other with prolonged decomposition time (Lecerf et al., 2011).

## 5 CONCLUSIONS

This study showed that litter mixtures had no significant effects on mass loss of most decomposing mixtures, implying that the influence of litter mixing on the decomposition process might be not common in this desert region. The N addition, however, did change the direction and magnitude of mixture effects, leading to suppression or stimulation of mixing effects. This result highlights that a future increase in N deposition will disrupt interactions within litter mixtures that modulate the decomposition process and, ultimately, affect C turnover in desert ecosystems. Additionally, incubation time plays an important role in mediating the influence of litter mixing on mass loss and its responses to N addition, which implies that short‐term experiments could underestimate the importance of litter diversity for decomposition under projected increasing N deposition. Thus, future long‐duration experiments are needed to deepen our understanding of general patterns of litter mixing decomposition under future climate change to accurately predict C budgets at the ecosystem level. Our results address the effects of interactions between litter mixing and N addition on litter mass loss and are crucial to inform future predictions of the responses in litter layers to changing N deposition in desert ecosystems.

## AUTHOR CONTRIBUTIONS

**Hong‐Mei Zhao:** Data curation (lead); funding acquisition (lead); methodology (lead); project administration (lead); writing – original draft (lead); writing – review and editing (lead). **Wei‐Jun Yang:** Investigation (equal); resources (equal); writing – original draft (supporting). **Jun‐Hui Cheng:** Formal analysis (supporting); funding acquisition (supporting). **Gang Huang:** Funding acquisition (supporting); project administration (supporting). **Yu‐Tong Hu:** Investigation (equal); resources (equal). **Cong‐Juan Li:** Funding acquisition (supporting); investigation (equal). **Jian‐Dong Sheng:** Conceptualization (lead); writing – review and editing (supporting).

## FUNDING INFORMATION

National Natural Science Foundation of China: 31700423, 32260280, 32171643, U1603235 the Funds for Distinguished Young of Xinjiang Natural Science Foundation: 2022D01E97.

## CONFLICT OF INTEREST STATEMENT

The authors have no conflict of interest.

## Data Availability

The data associated with this manuscript have been deposited in Dryad Digital Repository with https://doi.org/10.5061/dryad.05qfttf8c.
